# Aspects of the ecology of phlebotomine sand flies (Diptera: Psychodidae) in the Private Natural Heritage Reserve Sanctuary Caraça

**DOI:** 10.1371/journal.pone.0178628

**Published:** 2017-06-01

**Authors:** Gabriel Barbosa Tonelli, Aline Tanure, Felipe Dutra Rêgo, Gustavo Mayr de Lima Carvalho, Taynãna César Simões, José Dilermando Andrade Filho

**Affiliations:** 1 Grupo de Estudos em Leishmanioses, Centro de Pesquisas René Rachou, Fiocruz, Minas Gerais, Brasil; 2 Pesquisa Clínica e Políticas Públicas em Doenças Infecciosas e Parasitárias, René Rachou, Fiocruz, Minas Gerais, Brasil; Meharry Medical College, UNITED STATES

## Abstract

Leishmaniases are a set of parasitic diseases of zoonotic origin that are transmitted by sandfly vectors in wild, rural and urban environments. Their distribution is dependent not only the distribution of vectors, but also on the distribution of mammalian reservoirs. Only by understanding the transmission cycle of these diseases, such as knowing the participating vectors and reservoirs, can one can understand the epidemiology and ecological relationships of leishmaniases. Ecotourism has become an important area of economic growth in Brazil. One of the most visited tourist attractions in the state of Minas Gerais, the Reserva Particular do Patrimônio Natural Santuário do Caraça (RPPNSC) is located in the Quadrilátero Ferrífero. The aim of this study was to contribute to the control of leishmaniasis among tourists of the RPPNPC by surveying its sand fly fauna and testing for the presence of *Leishmania* DNA in females. Twenty-five CDC light traps were exposed on 7 trails of the RPPNPC where samples were collected bimonthly for a year, starting in June 2013. A total of 376 specimens of 18 species and 10 genera of sandflies were captured. The predominant species were *Psychodopygus lloydi* (72.34%) and *Pintomyia monticola* (5.59%). HaeIII restriction enzyme detected and characterized *Leishmania braziliensis* DNA in 2 of the samples for an infection rate of 0.7% (2/266). Recent studies found specimens of *Ps*. *lloyd* infected with *Leishmania braziliensis* elsewhere in Minas Gerais, which may be an indication that this species is involved in the transmission of *Leishmania* in this state.

## Introduction

Leishmaniases occur in about 100 countries in subtropical or tropical climates, and in anthroponotic and zoonotic cycles [1,2,3].

Leishmaniases are caused by 21 species of *Leishmania* and their epidemiology is known to involve several species of mammals, which act as reservoirs, and various species of sand flies, which act as vectors, of these protozoa, and so they are considered the most complex set of diseases transmitted by vectors [4,5,6]. The epidemiology of leishmaniases are only understood through knowledge of the links that make up their transmission cycle, such as the vectors and reservoirs involved and their ecological relationships [7]. *Lutzomyia longipalpis* (Lutz & Neiva, 1912), the main vector of *Leishmania infantum* in Brazil [8,9,10] is closely associated with birds and several species of domestic and synanthropic mammals, including humans who act as hosts and reservoirs. Other species, such as *Nyssomyia intermedia* (Lutz & Neiva, 1912) and *Nyssomyia whitmani* (Antunes & Coutinho, 1939) are important insect vector of *Leishmania braziliensis*, the etiological agent of cutaneous leishmaniasis (CL) in southeastern of Brazil. These three species of sand flies show a considerable degree of adaptation to altered environments and anthropophilic behavior regarding their food [11,12,13,14,15,16]. Other species of sand flies have been suspected of being vectors of *Leishmania*, for example, *Nyssomyia neivai* Pinto, 1926; *Evandromyia sallesi* Galvão & Coutinho, 1939; and *Psychodopygus lloydi* Antunes, 1937 [15].

The region of the Reserva Particular do Patrimônio Natural Santuário do Caraça (hereafter RPPNSC or Caraça Sanctuary) has experienced environmental pressure from neighboring municipalities where there have been autochthonous cases of human and canine leishmaniasis. The heavy movement of people and animals facilitates contact between vector and reservoir, and vector and humans, which could result in an increase in the incidence of infection and the consequent increase in the number of cases of disease. Caraça Sanctuary receives an average of 60,000 visitors per year, of which at least 17,500 are guests in their facilities, making it one of the most important and most visited Conservation Units in the state of Minas Gerais [17].

This paper aims to describe the patterns of species richness and diversity of sandflies among areas of Caraça Sanctuary and to investigate their seasonal variation. It also aims to assess the presence of *Leishmania* DNA among these insects.

## Materials and methods

### Study area

**Fig 1:**
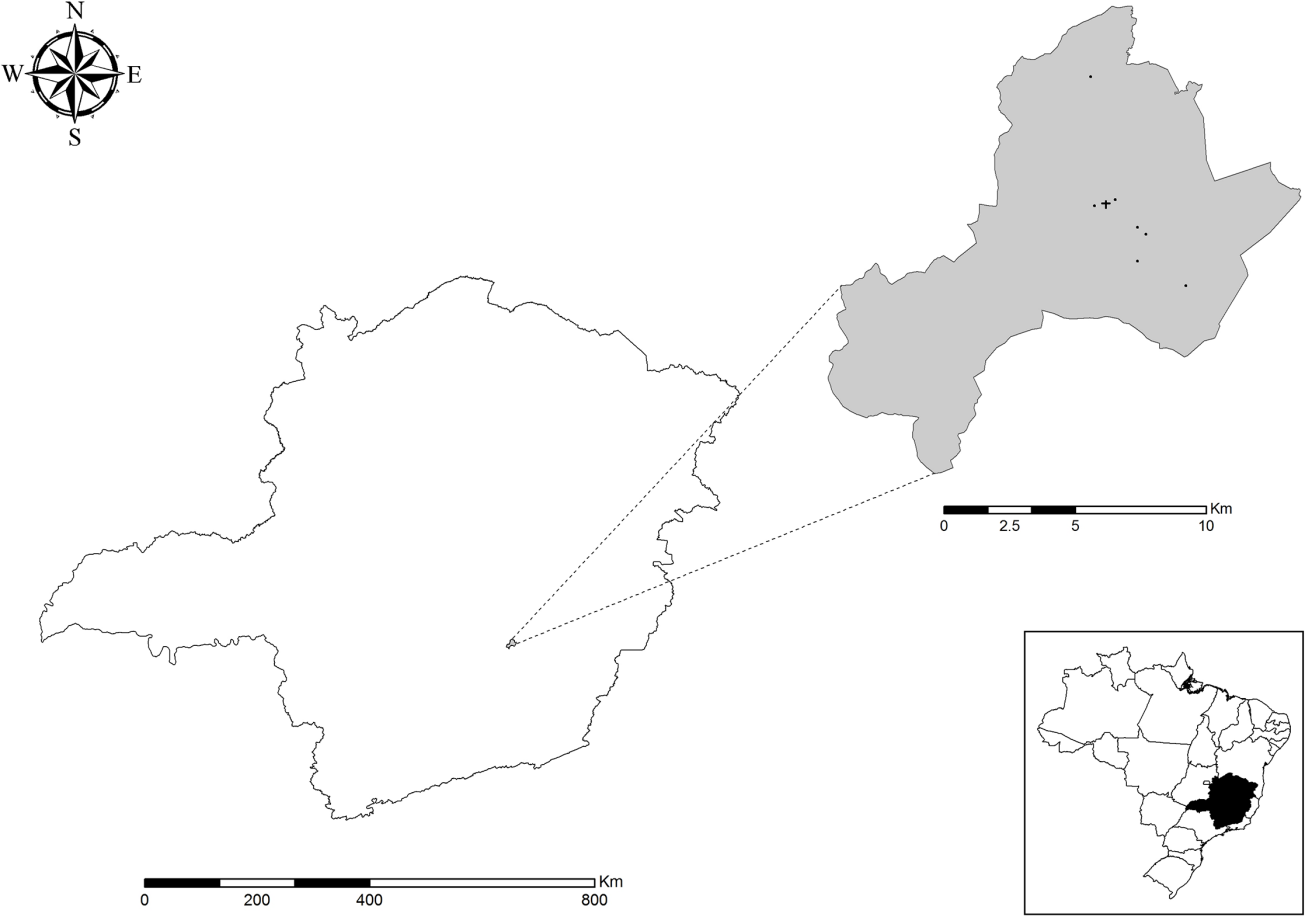
Location of the RPPN Santuário do Caraça on the state of Minas Gerais, Brazil and distribution of sample sites among the RPPN area. Black dots represents the sample sites and the black cross it’s the location of the head office of the RPPN.

Caraça Sanctuary is located in the municipalities of Santa Barbara, Barão de Cocais and Catas Altas (Fig 1), in the state of Minas Gerais, Brazil (20°0′51″ S, 43°29′28″ W). It encompasses an area of 10,187.89 hectares with a maximum altitude of 2,072 meters above the sea level at “Pico do Sol” [17].

Caraça Sanctuary is situated on the slopes of the “Serra do Espinhaço”, mountain range and possesses a variety of floras including semideciduous forests (Atlantic Forest), savannah (Cerrado), and open areas such as high-altitude and rocky (rupestrian) fields. The annual minimum and maximum temperatures are 7°C and 30°C, respectively, although on rare occasions it gets below 0°C [18].

### Collection of sand flies

All the collection was carried out in accordance with the Permanent License to Collect Zoologic Materials n° 15237–2 of Ministério do Meio Ambiente–MMA (File 1).

**Fig 2:**
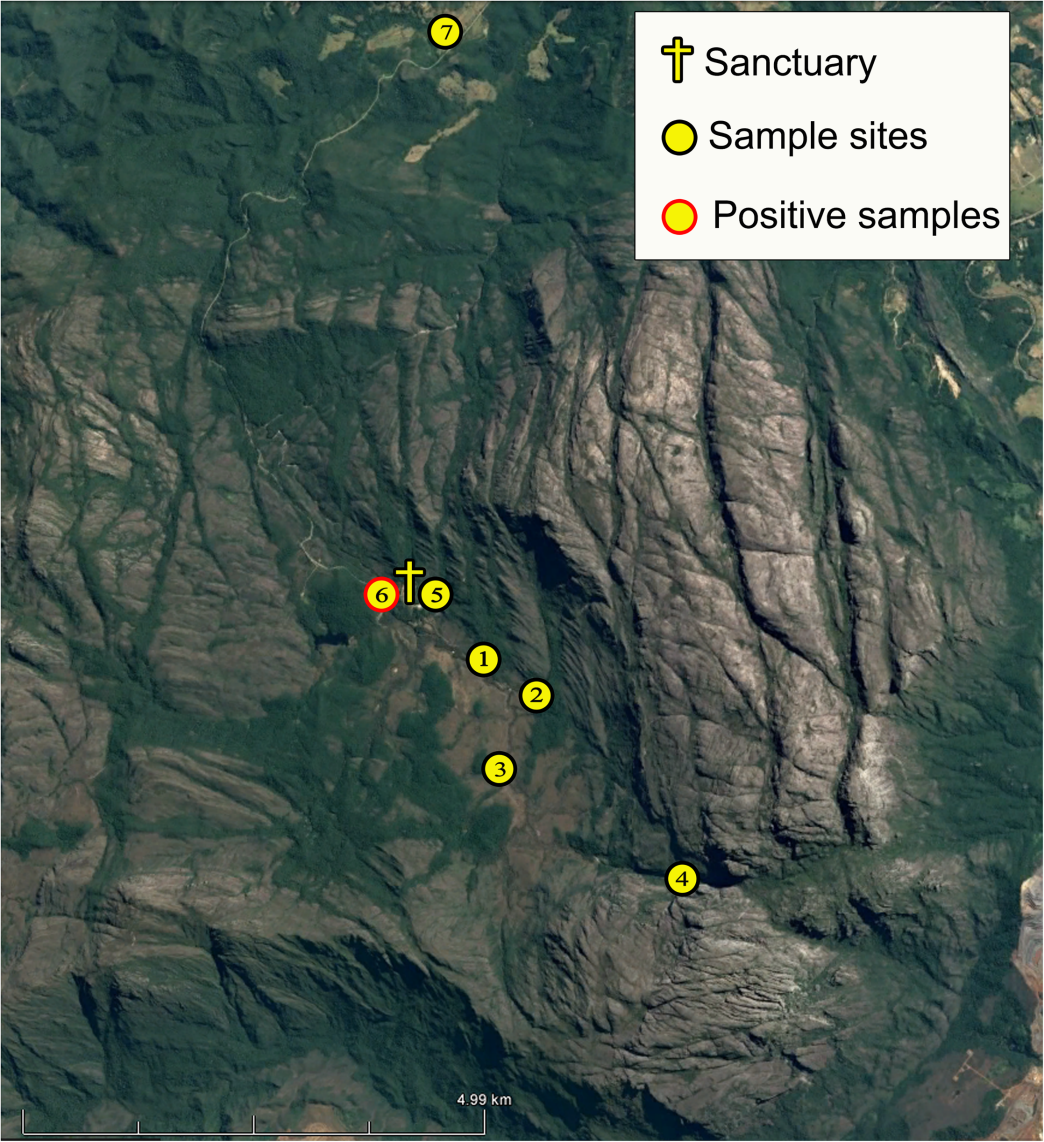
Sand fly sample sites and location site where DNA of *Le*. *braziliensis* was detected by PCR CYTb in two specimens of *Ps*. *lloydi* on the RPPN Santuário do Caraça. The red border in the yellow dot represents the location where the specimens of *Ps*. *lloydi* sand flies where detected with *Le*. *braziliensis* DNA.

We used 25 CDC light traps distributed among seven trails in the RPPNSC. Trails 1 and 2 were placed in forested areas; trails 3 and 4 in rupestrian fields (ecotope of Cerrado biome) and in one cave; trails 5 and 6 in peridomestic and intradomestic areas (house provided for researchers); and trail 7 in the peridomicile area of the hotel (Fig 2). Bimonthly systematic sampling was performed between June 2013 and June 2014. The temperature and relative humidity were measured during the weeks of trapping with the aid of an analog thermometer.

Sand flies were stored in labeled tubes containing 70% alcohol for subsequent analysis and identification. In the lab, males were mounted in Berlese liquid for species identification while females, which were identified by the structure of the cibarium and presence of spermathecae, were dissected for subsequent molecular analysis. The classification used was that proposed by Galati [19]. Voucher species were deposited in the “Coleção de Flebotomíneosb do Centro de Pesquisas René Rachou/Fiocruz (FIOCRUZ-COLFLEB” (Anexo 2).

### Dna extraction and *Leishmania* identification

DNA extraction was performed for individual female sand flies using Genra Puregene Kit (Qiagen, USA) following the manufacturer's protocol.

The extracted DNA was subjected to molecular analysis for the 300–350 bp amplification fragment of the intergenic region of *Leishmania* DNA (Internal Transcribed Spacer q—ITS1) using the primers LITSR: 5´ CTGGATCATTTTCCGATG 3´ and L5.8S: 5´ TGATACCACTTATCGCACTT 3.

DNA extracted from the strains of *Leishmania amazonensis* (IFLA/BR/67/PH8), *Le*. *braziliensis* (MHOM/BR/75/M2903), *Le*. *infantum* (MHOM/BR/74/PP75) and *Le*. *guyanensis* (MHOM/BR/75/M4147) were used as positive controls for the PCR. The amplified product was subjected to electrophoresis in a 2% agarose gel, which was then stained with ethidium bromide (7mg / mL) with molecular weight of 100pb.

For identification of *Leishmania* species, the amplified product of PCR ITS1 (10-15uL) was digested using HaeIII enzyme (10U / uL), according to the manufacturer's recommendations (New England Biolabs, Ipswich, MA, USA). The restriction patterns were analyzed on a 4% agarose gel stained with ethidium bromide (7mg / mL) in comparison with the reference strains of *Leishmania* mentioned above.

### Statistical analysis

We use the index of species abundance (ISA) and, in sequence, the standardized index of species abundance [20] to assess species abundance in the study area. The values for SISA vary from 0 to 1, with 1 representing the highest abundance. To assess the diversity of species and the uniformity of abundance among collection sites we used the indices of Shannon (H) and Evenness (J) [21] respectively. To assess temporal variation and any association between the number of sandflies collected and climatic variables at RPPNSC between June 2013 and June 2014 we used generalized linear models (GLM) in which the probability distribution was the Binomial Negative, in order to consider the overdispersion of the number of sand flies. The offset term was the natural logarithm of the number of traps observed at each collection time. The temporal tendency was evaluated by a linear temporal term in the model. Descriptive analysis of the data was performed using Microsoft Excel (Office 2010). Statistical analyses were performed with the aid of the statistical software R (R Development Core Team, 2015).

## Results

A total of 376 sand flies were collected, of which 300 were females and 76 males, representing 18 species of 10 genera. The most representative genera were *Evandromyia* and *Psychodopygus*, with four species each. The species with the highest prevalence was *Psychodopygus lloydi* (Antunes, 1937) (72.79%), followed by *Brumptomyia troglodytes* (Lutz, 1922) (5.25%), *Nyssomyia whitmani* (4.01%) and *Pintomyia monticola* (Costa Lima, 1932) (4.30%). The indices of Shannon (H) and Evenness (J) were low (H’ = 1.23; J’ = 0.43). According to the SISA, the most abundant species were *Br*. *troglodytes* (0.52) *Ps*. *lloydi* (0.39) and *Mi*. *ferreirana* (0.35), whereas the least abundant species were *Ev*. *termitophila* (0.03) and *Lu*. *longipalpis* (0.07). Except in Gruta da Bocaina, *Ps*. *lloydi* was captured among all trails. The most productive sampling points were Engenho (27.19%), Casa das Sampaias (24.27%) and Mata Cascatinha (24.27%) (Table 1).

**Table 1:** Sand Flies collected on RPPN Santuário do Caraça. Trail 1 –Mata da Cascatinha, Trail 2 –Cascatinha, Trail 3 –Pedra da Paciência, Trail 4 –Gruta da Bocaina, Trail 5 –Casa dos Pesquisadores, Trail 6 –Casa das Sampaias, Trail 7 –Engenho.

Species/Trails	1		2		3		4		5		6		7		TOTAL		%
* *	♂	♀	♂	♀	♂	♀	♂	♀	♂	♀	♂	♀	♂	♀	♂	♀	
*Brumptomyia troglodytes*	7	4	3	0	1	0	0	0	1	1	2	1	0	0	**14**	**6**	5,25%
*Evandromyia evandroi*	0	0	0	0	0	0	0	0	0	0	0	0	0	1	**0**	**1**	0,27%
*Evandromyia lenti*	0	0	1	0	0	0	0	0	0	0	0	0	8	3	**9**	**3**	3,21%
*Evandromyia termitophila*	0	0	0	0	0	0	0	0	0	0	0	0	0	4	**0**	**4**	1,07%
*Evandromyia tupynambai*	0	2	0	0	0	0	0	0	0	0	0	1	0	0	**0**	**3**	0,80%
*Lutzomyia ischyracanta*	0	0	1	0	0	0	0	0	0	0	0	0	0	0	**1**	**0**	0,27%
*Lutzomyia longipalpis*	0	0	0	0	0	0	0	0	0	0	0	0	0	2	**0**	**2**	0,53%
*Micropigomyia ferreirana*	0	2	1	0	0	0	0	0	0	0	1	0	0	0	**2**	**2**	0,80%
*Nyssomyia whitmani*	0	0	0	0	2	1	2	4	1	0	0	0	1	5	**6**	**10**	4,01%
*Pintomyia misionensis*	1	0	0	0	0	0	0	0	0	0	0	0	0	0	**1**	**0**	0,27%
*Pintomyia monticola*	6	4	0	1	0	0	0	0	0	0	0	0	0	5	**6**	**10**	4,30%
*Psathyromyia pestanai*	1	0	6	0	0	0	0	0	0	0	0	0	0	0	**7**	**0**	1,87%
*Psychodopygus ayrozai*	1	0	0	0	0	0	0	0	0	0	0	0	0	0	**1**	**0**	0,27%
*Psychodopygus carrerai*	0	0	0	0	0	0	0	0	0	0	0	0	0	1	**0**	**1**	0,27%
*Psychodopygus lloydi*	5	37	4	20	1	9	0	0	3	44	4	78	0	67	**17**	**255**	72,79%
*Psychodopygus pascalei*	9	3	0	0	0	0	0	0	0	0	1	0	0	0	**10**	**3**	3,48%
*Sciopemyia sordellii*	0	0	0	0	0	0	0	0	0	0	0	0	1	0	**1**	**0**	0,27%
*Trichopygomyia longispina*	0	0	0	0	0	0	0	0	0	0	0	0	1	0	**1**	**0**	0,27%
**TOTAL**	82 (24,27%)		37 (9,36%)		14 (3,22%)		6 (1,75%)		50 (9,94%)		88 (24,27%)		99 (27,19%)		**76**	**300**	
* *															**376**		

**Fig 3:**
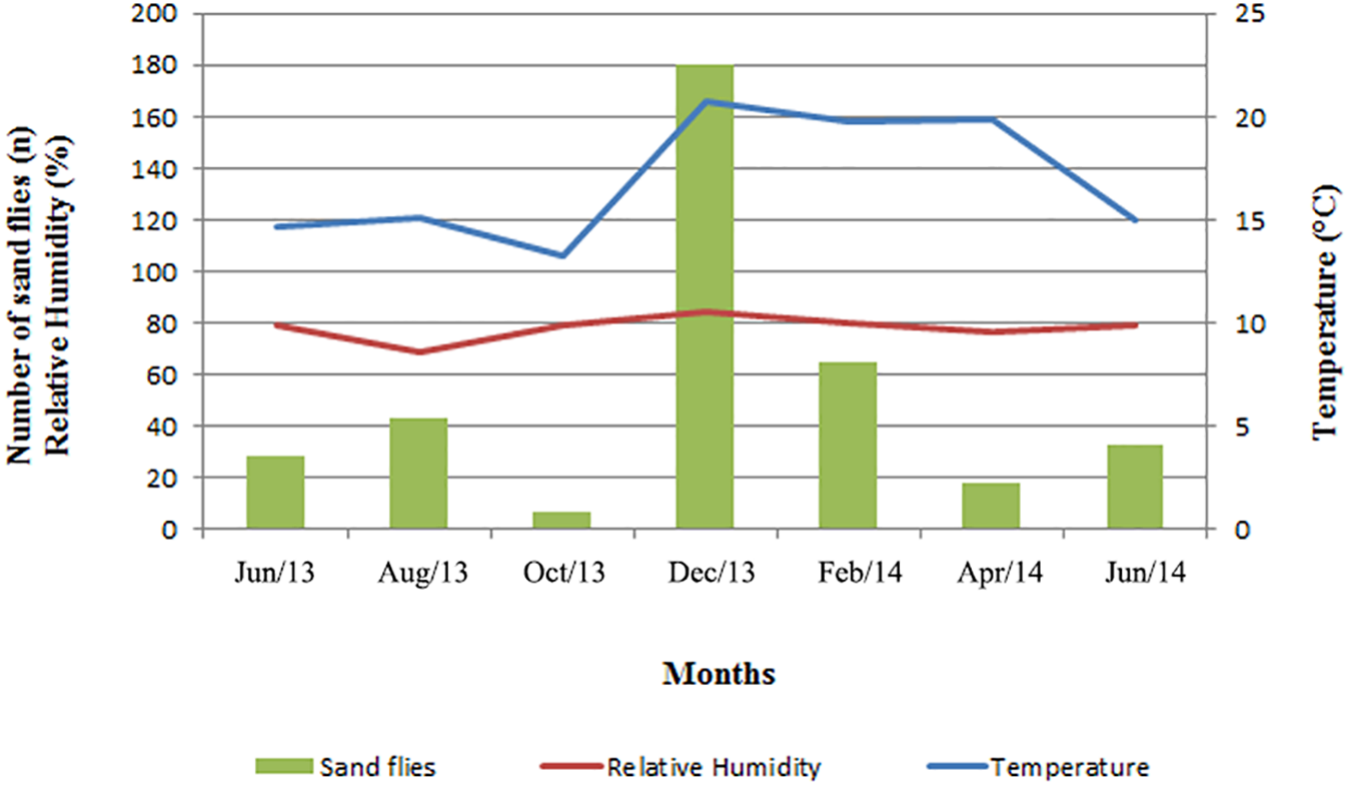
Sazonality of Sand Flies caught in the RPPN Santuário do Caraça between June 2013 to July 2014. The red line represents the variation of the relative humidity during the collection period and the blue line represents the variation of the temperature during the sampling period.

The months with the greatest sampling success were August 2013 (12%), December 2013 (48%) and February 2014 (17%), whereas those with the least success were June 2013 (7%), October 2013 (2%) and April 2014 (5%). However, the model did not show significant variability in the linear temporal term, thus, the number of sand flies was considered constant over time (p-value = 0.862). Local temperatures demonstrated low values during the collection and the average of the coldest months 14.7°C on June 2013 and 13.23°C on October 2013 and the average of the hottest months was 20.75°C on December 2013 and 19.76°C on February 2014. The model showed a significant variation of 1.2 in the average number of sand flies with the increase of 1 degrees of Celsius in the temperature (p-value < 0.01). Monthly averages of relative humidity remained above 60%, with the highest being in December 2013 (84.38%), followed by February 2014 (79.8%), whereas those with the lowest were August 2013 (69.13%) and April 2014 (76.8%), so that variation was not significant (p-value = 0.460) (Fig 3).

**Fig 4:**
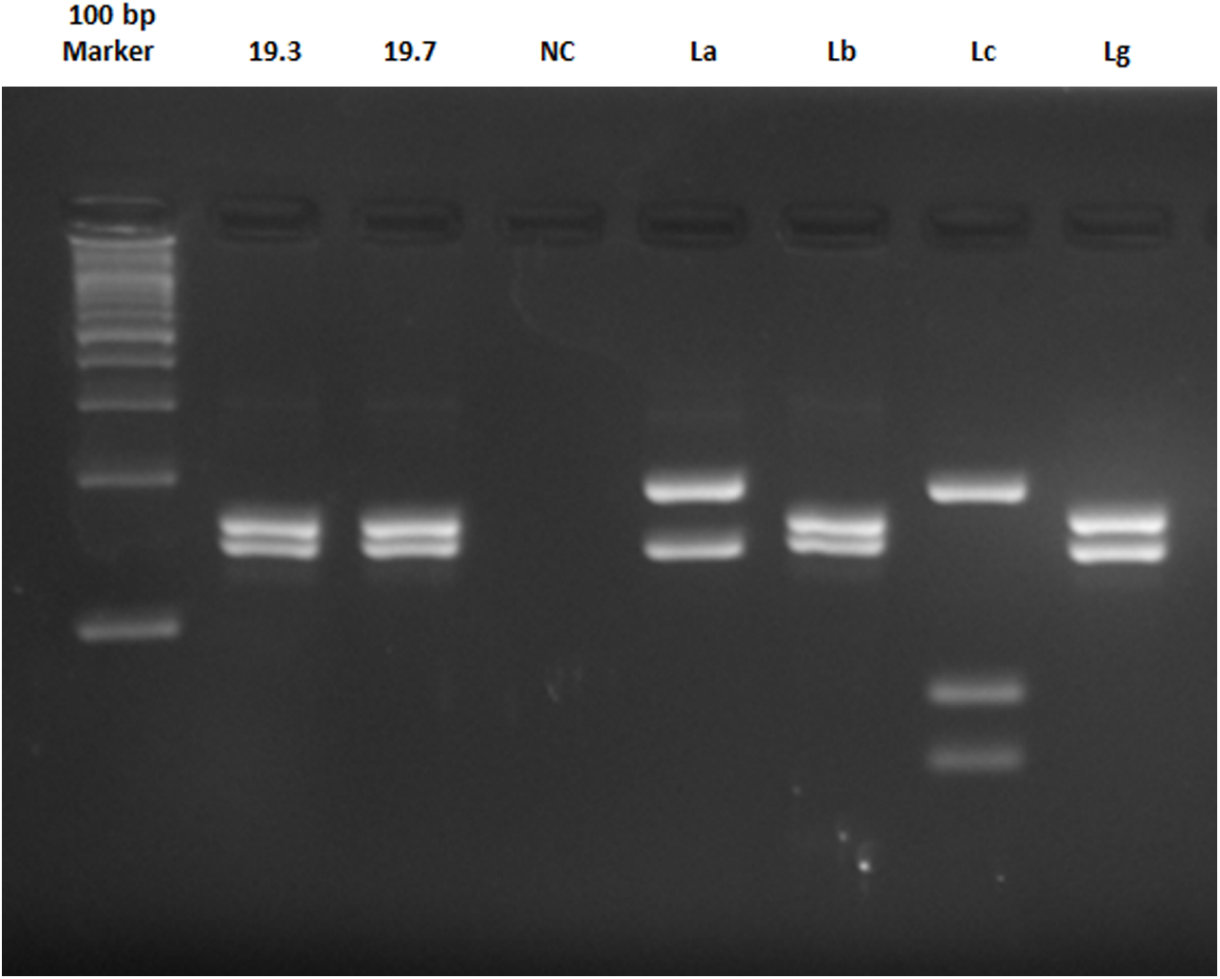
Electrophoresis 4% agarose gel of the RFLP ITS1 of positive DNA samples of sand flies collected in RPPNSC. MW = Molecular Weight, 19.3 and 19.7 = samples, CN = Negative Control, La, Lb, Lc and Lg = Positive Controls strains of *Leishmania amazonensis*, *Le*. *braziliensis*, *Le*. *infantum* and *Le*. *guyanensis* respectively.

Two of the 300 samples analyzed had 300–350 bp fragments detected by PCR of DNA ITS1 extracted from sand flies, indicating a positive result for the presence *Leishmania* DNA. Species identification using PCR-RFLP indicated the profile of *Le*. *braziliensis* in both of the positive samples (Fig 4). Both samples were from individuals of *Ps*. *lloydi*.

## Discussion

Leishmaniases have complex relationships with their vectors and reservoirs, and so their ecology makes understanding these diseases challenging. Several mammalian species with different behaviors can act as reservoirs, as is also true for sandfly vectors and vector competence [22,23]. Furthermore, different parasites use different defense mechanisms against the immune system of reservoirs and vectors, which favor infection [24].

It is important to point out that the municipalities surrounding the RPPNSC possess high densities of *Lu*. *longipalis* and *Ny*. *Whitmani*, such as has been found in Barão de Cocais and Catas Altas (unpublished data). Furthermore, surveys done in collaboration with the health departments of each municipality detected autochthonous cases of human and canine leishmaniasis at three sites, including human cases of visceral and cutaneous leishmaniasis and one death.

Despite there never having been a diagnosed case of leishmaniasis in RPPNSC, it is possible that it exerts pressure on nearby locations with regard to parasitic diseases that depend on vectors and reservoirs that are present in its extensive preserved forest and among the diversity of its fauna. Some mammalian species have been reported in the study area [18] that may have an important role in the maintenance of the *Leishmania* transmission cycle, since they can serve as reservoirs for this multi-reservoir parasite [25,26,7,27].

The sand fly species observed in this study comprise a fauna similar to that found in other studies carried out in nature reserves as well as in wild areas. The great diversity of species collected in these environments represent a different profile than that observed in urban areas [28,29,30].

In Ibitipoca State Park, a region with climatic and topographic characteristics similar to those of RPPNSC (high humidity, high altitudes and low temperatures), Carvalho [31] sampled some of the same species as the present study, including *Ps*. *lloydi*, *Psychodopygus pascalei* (Coutinho & Barretto, 1940), *Pi*. *monticola*, *Evandromyia Lenti* (Mangabeira, 1938) and *Br*. *Troglodytes*. These local climatic factors may explain the lower number of individuals collected in relation to other that found in other studies of sandfly faunas [32,33].

Some of species found in this study at RPPNSC have been incriminated as potential vectors of *Leishmania*, such as *Lu*. *longipalpis* and *Ny*. *whitmani* [34,35,36,37,38,39,4,40,41,42,43,44,45,46,47]. Others have been detected with *Leishmania* DNA, such as *Evandromyia Lenti*, *Evandromyia termitophila* (Martins, Falcão & Silva, 1964), *Micropygomyia ferreirana* (Barreto, Martin & Pellegrino, 1956) and *Ps*. *Lloydi* [48,49,50,51,52,36,53,54,55,56,57], which may suggest they play a role in maintaining the parasite transmission cycle in wild environments.

The most abundant species captured in the present study was *Ps*. *lloydi*, which possesses a wide geographical distribution that encompasses the states of Minas Gerais, Maranhão, Paraná, Rio de Janeiro and São Paulo [58,59,60]. According to Santos [59], most species of the genus *Psychondopygus* occur only in wild habitats, and Rangel & Lainson [61], explain that some species of the genus are important in the transmission of cutaneous leishmaniasis, such as *Psychodopygus wellcomei* (Fraiha, Shaw & Lainson 1971), complex *Psychodopygus* (Mangabeira, 1941), *Psychodopygus paraensis* (Costa Lima, 1941) and *Psychodopygus ayrozai* (Barretto & Coutinho, 1940).

Two approaches have been used to diagnose *Leishmania* infection of, or the presence of *Leishmania* DNA in, vectors. The traditional gold standard for diagnosing this disease has been the dissection of female sand flies. This technique has the advantage of permitting the observation of the parasites flagella and its shape and position in the insect's digestive tract, however, skilled labor is necessary and yet it is still time consuming, with a lot of specimens needing to be analyzed in order to obtain meaningful data (as discussed by Brazil and Brazil [62]. Moreover, this method does not permit the identification to genus and species, which requires isolation or molecular analysis of the parasite for identification, because trypanosomatides other than *Leishmania* may be found in sandflies [63,64,65,66].

The second approach for diagnosis of infection or DNA detection is the use of molecular techniques, such as PCR, with different targets because it is very sensitive and highly specific, which is important with *Leishmania* because the infection rate of vectors is relatively low [67]. Some targets used to analyze infection in sandflies are kDNA-PCR [68], Real Time PCR [69] and ITS1 [54].

In our study, two females of the species *Ps*. *lloydi* tested positive for DNA of *Le*. *braziliensis* ITS1 by RFLP-PCR, for an infection rate of 0.6% (2/300). These two specimens were captured in December 2013. This species was also found infected by Quaresma [54], who suggests that it may play a role as a vector in the sylvatic cycle of *Le*. *braziliensis* in this environment since this is the most abundant species found in the work and it was captured in every month of sampling. The species *Ps*. *lloydi* and *Pintomyia monticola* (Costa Lima, 1932) were the most abundant species in a collection carried out with Shannon traps (unpublished data), which reinforces the epidemiological importance of these species in relation to maintaining the sylvatic cycle of *Le*. *braziliensis*.

It is useful to highlight the importance of additional data since this species can feed on several mammals (rodents / marsupials) [54], which have been found to play important roles as hosts / *Leishmania* reservoirs in other studies [27,70,71]. The mammal fauna of Caraça Sanctuary is considered to be diverse [18], and so several species could act as reservoirs and parasite hosts and maintaining the sylvatic cycL.

Both sand flies infected with *Le*. *braziliensis* DNA were captured on the trail of the Casa das Sampaias, a place with a high frequency of tourist visits. In environments where vectors are found that may have anthropophilic behavior, potential reservoirs and etiological agents are deserving of special attention in relation to risk of ACL transmission. In the case of RPPNSC, which receives many visitors from various locations around the world each year, the possibility exists that leishmaniasis could migrate through travelers, an issue already mentioned by Antinori et al [72] and of increasing concern to travelers visiting environments where there is a focus.

Based on the criteria for incriminating a vector of Killick-Kendrick & Ward [23], which consider the distribution of the analyzed species and its abundance in the place of study are important. The months of December to February at Caraça Sanctuary exhibited climatic conditions with high temperature and humidity, and are also the months in which we observed higher abundances of sand flies. This illustrates the direct relationship between temperature and the number of captured sandflies, as seen in Fig 1, since every 1°C increase the average of individuals caught per trap according to temporal analysis negative binomial. The individual sandflies of *Ps*. *lloydi* that tested positive in the ITS PCR—RFLP were captured on December 2013, based on it’s local abundance and distribution, suggesting that this period may be wild infection and that this time occurs cycle maintain on this environment making this period of most epidemiological attention regarding the transmission of leishmaniasis in place.

The data here, combined with vector control efforts, can strengthen the sandfly management plan of Santuário do Caraça. Studies of infection in local mammals, and other fauna, are of great importance for determining hosts / reservoirs and understanding *Leishmania* circulation.

## Supporting information

S1 File Permanent License to Collect Zoologic Materials n° 15237–2 of Ministério do Meio Ambiente–MMA. (PDF)
