# Supplementary material for: Aspects of the ecology of phlebotomine sand flies (Diptera: Psychodidae) in the Private Natural Heritage Reserve Sanctuary Caraça
Source: PLoS One. 2017 Jun 1;12(6):e0178628. doi: 10.1371/journal.pone.0178628 (PMC5453570; doi:10.1371/journal.pone.0178628)
Supplement: S1 File — (PDF) [file pone.0178628.s001.pdf]

Ministério do Meio Ambiente - MMA
Instituto Chico Mendes de Conservação da Biodiversidade - ICMBio
Sistema de Autorização e Informação em Biodiversidade - SISBIO

Licença permanente para coleta de material zoológico

**Número: 15237-2**	**Data da Emissão: 29/11/2011 17:39**

Dados do titular

Nome: José Dilermando Andrade Filho	CPF: 835.584.546-34
Nome da Instituição : CENTRO DE PESQUISAS RENÉ RACHOU-FIOCRUZ	CNPJ: 33.781.055/0008-01

Observações e ressalvas

1	As atividades de campo exercidas por pessoa natural ou jurídica estrangeira, em todo o território nacional, que impliquem o deslocamento de recursos humanos e materiais, tendo por objeto coletar dados, materiais, espécimes biológicos e minerais, peças integrantes da cultura nativa e cultura popular, presente e passada, obtidos por meio de recursos e técnicas que se destinem ao estudo, à difusão ou à pesquisa, estão sujeitas a autorização do Ministério de Ciência e Tecnologia.
2	A licença permanente não é válida para: a) coleta ou transporte de espécies que constem nas listas oficiais de espécies ameaçadas de extinção; b) manutenção de espécimes de fauna silvestre em cativeiro; c) recebimento ou envio de material biológico ao exterior; e d) realização de pesquisa em unidade de conservação federal ou em caverna. A restrição prevista no item d não se aplica às categorias Reserva Particular do Patrimônio Natural, Área de Relevante Interesse Ecológico e Área de Proteção Ambiental constituídas por terras privadas.
3	O pesquisador titular da licença permanente, quando acompanhado, deverá registrar a expedição de campo no Sisbio e informar o nome e CPF dos membros da sua equipe, bem como dados da expedição, que constarão no comprovante de registro de expedição para eventual apresentação à fiscalização;
4	Esta licença permanente NÃO exime o pesquisador titular da necessidade de obter as anuências previstas em outros instrumentos legais, bem como do consentimento do responsável pela área, pública ou privada, onde será realizada a atividade, inclusive do órgão gestor de terra indígena (FUNAI), da unidade de conservação estadual, distrital ou municipal.
5	Esta licença permanente não poderá ser utilizada para fins comerciais, industriais ou esportivos ou para realização de atividades integrantes do processo de licenciamento ambiental de empreendimentos.
6	Este documento NÃO exime o pesquisador titular da necessidade de atender ao disposto na Instrução Normativa Ibama nº 27/2002, que regulamenta o Sistema Nacional de Anilhamento de Aves Silvestres.
7	O pesquisador titular da licença permanente será responsável pelos atos dos membros da equipe (quando for o caso)
8	O órgão gestor de unidade de conservação estadual, distrital ou municipal poderá, a despeito da licença permanente e das autorizações concedidas pelo ICMBio, estabelecer outras condições para a realização de pesquisa nessas unidades de conservação.
9	O titular de licença ou autorização e os membros da sua equipe deverão optar por métodos de coleta e instrumentos de captura direcionados, sempre que possível, ao grupo taxonômico de interesse, evitando a morte ou dano significativo a outros grupos; e empregar esforço de coleta ou captura que não comprometa a viabilidade de populações do grupo taxonômico de interesse em condição in situ.
10	O titular da licença permanente deverá apresentar, anualmente, relatório de atividades a ser enviado por meio do Sisbio no prazo de até 30 dias após o aniversário de emissão da licença permanente.
11	O titular de autorização ou de licença permanente, assim como os membros de sua equipe, quando da violação da legislação vigente, ou quando da inadequação, omissão ou falsa descrição de informações relevantes que subsidiaram a expedição do ato, poderá, mediante decisão motivada, ter a autorização ou licença suspensa ou revogada pelo ICMBio e o material biológico coletado apreendido nos termos da legislação brasileira em vigor.
12	A licença permanente será válida enquanto durar o vínculo empregatício do pesquisador com a instituição científica a qual ele estava vinculado por ocasião da solicitação.
13	Este documento não dispensa o cumprimento da legislação que dispõe sobre acesso a componente do patrimônio genético existente no território nacional, na plataforma continental e na zona econômica exclusiva, ou ao conhecimento tradicional associado ao patrimônio genético, para fins de pesquisa científica, bioprospecção e desenvolvimento tecnológico. Veja maiores informações em www.mma.gov.br/cgen.
14	As atividades contempladas nesta autorização NÃO abrangem espécies brasileiras constante de listas oficiais (de abrangência nacional, estadual ou municipal) de espécies ameaçadas de extinção, sobreexplotadas ou ameaçadas de sobreexplotação.

Táxons autorizados

#	Nível taxonômico	Táxon(s)
1	FAMILIA	Psychodidae
2		

Destino do material biológico coletado

#	Nome local destino	Tipo Destino
1	UFMG - UNIVERSIDADE FEDERAL DE MINAS GERAIS	coleção
2	CENTRO DE PESQUISAS RENÉ RACHOU-FIOCRUZ	coleção

Este documento (Licença permanente para coleta de material zoológico) foi expedido com base na Instrução Normativa nº154/2007. Através do código de autenticação abaixo, qualquer cidadão poderá verificar a autenticidade ou regularidade deste documento, por meio da página do Sisbio/ICMBio na Internet (www.icmbio.gov.br/sisbio).

Código de autenticação: 36646273

Licença permanente para coleta de material zoológico

**Número: 15237-2**	**Data da Emissão: 29/11/2011 17:39**

Dados do titular

Nome: José Dilermando Andrade Filho	CPF: 835.584.546-34
Nome da Instituição : CENTRO DE PESQUISAS RENÉ RACHOU-FIOCRUZ	CNPJ: 33.781.055/0008-01

Registro de coleta imprevista de material biológico

De acordo com a Instrução Normativa nº154/2007, a coleta imprevista de material biológico ou de substrato não contemplado na autorização ou na licença permanente deverá ser anotada na mesma, em campo específico, por ocasião da coleta, devendo esta coleta imprevista ser comunicada por meio do relatório de atividades. O transporte do material biológico ou do substrato deverá ser acompanhado da autorização ou da licença permanente com a devida anotação. O material biológico coletado de forma imprevista, deverá ser destinado à instituição científica e, depositado, preferencialmente, em coleção biológica científica registrada no Cadastro Nacional de Coleções Biológicas (CCBIO).

Táxon*	Qtde.	Tipo de amostra	Qtde.	Data

* Identificar o espécime no nível taxonômico possível.

Este documento (Licença permanente para coleta de material zoológico) foi expedido com base na Instrução Normativa nº154/2007. Através do código de autenticação abaixo, qualquer cidadão poderá verificar a autenticidade ou regularidade deste documento, por meio da página do Sisbio/ICMBio na Internet (www.icmbio.gov.br/sisbio).

Código de autenticação: 36646273
